# Severe Psychological Distress of Evacuees in Evacuation Zone Caused by the Fukushima Daiichi Nuclear Power Plant Accident: The Fukushima Health Management Survey

**DOI:** 10.1371/journal.pone.0158821

**Published:** 2016-07-08

**Authors:** Yasuto Kunii, Yuriko Suzuki, Tetsuya Shiga, Hirooki Yabe, Seiji Yasumura, Masaharu Maeda, Shin-ichi Niwa, Akira Otsuru, Hirobumi Mashiko, Masafumi Abe, Mental Health Group of the Fukushima Health Management Survey

**Affiliations:** 1 Department of Neuropsychiatry, School of Medicine, Fukushima Medical University, Fukushima, Japan; 2 Department of Adult Mental Health, National Institute of Mental Health, National Center of Neurology and Psychiatry, Tokyo, Japan; 3 Radiation Medical Science Center for the Fukushima Health Management Survey, Fukushima Medical University, Fukushima, Japan; 4 Department of Public Health, School of Medicine, Fukushima Medical University, Fukushima, Japan; 5 Department of Disaster Psychiatry, School of Medicine, Fukushima Medical University, Fukushima, Japan; 6 Department of Psychiatry, Aizu Medical Center, Fukushima Medical University, Fukushima, Japan; 7 Department of Radiation Health Management, School of Medicine, Fukushima Medical University, Fukushima, Japan; University of Jyväskylä, FINLAND

## Abstract

**Background:**

Following the Great East Japan Earthquake on March 11, 2011, the nuclear disaster at the Fukushima Daiichi Nuclear Power Plant has continued to affect the mental health status of residents in the evacuation zone. To examine the mental health status of evacuee after the nuclear accident, we conducted the Mental Health and Lifestyle Survey as part of the ongoing Fukushima Health Management Survey.

**Methods:**

We measured mental health status using the Kessler 6-item psychological distress scale (K6) in a total of 73,569 (response rate: 40.7%) evacuees aged 15 and over who lived in the evacuation zone in Fukushima Prefecture. We then dichotomized responders using a 12/13 cutoff on the K6, and compared the proportion of K6 scores ≥13 and ≤12 in each risk factor including demographic information, socioeconomic variables, and disaster-related variables. We also performed bivariate analyses between mental health status and possible risk factors using the chi-square test. Furthermore, we performed multivariate regression analysis using modified Poisson regression models.

**Results:**

The median K6 score was 5 (interquartile range: 1–10). The number of psychological distress was 8,717 (14.6%). We found that significant differences in the prevalence of psychological distress by almost all survey items, including disaster-related risk factors, most of which were also associated with increased Prevalence ratios (PRs). Additionally, we found that psychological distress in each evacuation zone was significantly positively associated with the radiation levels in their environment (*r* = 0.768, *p* = 0.002).

**Conclusion:**

The earthquake, tsunami and subsequent nuclear accident likely caused severe psychological distress among residents in the evacuation zone in Fukushima Prefecture. The close association between psychological distress and the radiation levels shows that the nuclear accident seriously influenced the mental health of the residents, which might be exacerbated by increased risk perception. To provide prompt and appropriate support, continued psychosocial intervention for evacuees is strongly recommended.

## Introduction

The Great East Japan Earthquake occurred on March 11, 2011, with a recorded magnitude of 9.0 on the Richter scale [1]. The epicenter was almost 130 km southeast of Oshika Peninsula, Miyagi Prefecture, and the resultant tsunami led to the accident at the Fukushima Daiichi Nuclear Power Plant, which was eventually classified by the International Atomic Agency as a Level 7 nuclear accident on the International Nuclear Event Scale [2]. This accident is an unprecedented nuclear disaster, with dispersion of radioactive material over wide areas of Fukushima Prefecture likely to continue over the long term. Ultimately, over 18,000 were killed or are still considered missing, and more than 399,000 houses were completely or partially destroyed by the earthquake and tsunami [3]. The numbers of deaths caused by the tsunami in Fukushima Prefecture were relatively lower compared with those in Iwate and Miyagi Prefectures, but the numbers of evacuees from Fukushima mainly caused by the nuclear power plant accident were greater than those in other prefectures [3]. Although the number of evacuees from Fukushima Prefecture steadily decreased from 164,865 recorded in May 2012 to 116,284 noted in March 2015, even now many citizens of Fukushima are not permitted to return to their homes. Currently, there are approximately 69,000 evacuees living in and estimated 47,000 living outside Fukushima Prefecture[4].

Looking at past nuclear disasters, a major problem indicated as a long-term health effect from the Chernobyl nuclear accident is psychosomatic abnormalities[5]. In other words, anxieties over radiation, unexplained physical symptoms, and subjective health concerns have all been identified among residents in exposed areas[6]. Even after this disaster, the World Health Organization has cited mental health as a major challenge [7, 8]. The earthquake and tsunami in Fukushima Prefecture left residents severely traumatized, and the sustained leakage of radioactive material due to a succession of accidents at the Fukushima Daiichi Nuclear Power Plant may be a significant source of fear and anxiety. In fact, as reported even in studies that pre-date the Great East Japan Earthquake, the Japanese are highly cognizant of the risks of nuclear accidents[9]. The stress felt by the citizens of Fukushima Prefecture, as they continue to feel the effects of the accident at the nuclear power plant that have followed even without adequate post-quake recovery, is of a type that we could not imagine before this disaster. Therefore, at Fukushima Medical University, in order to provide adequate care and gain a sense of the degree of prefectural residents’ mental health and lifestyles, we have implemented a Mental Health and Lifestyle Survey as part of the ongoing Fukushima Health Management Survey.

Until now, no large-scale systematic survey regarding the mental health status of evacuees from the Fukushima Daiichi Nuclear Power Plant accident has been reported, although a few studies on changes in conditions among psychiatric patients following the nuclear accident have been conducted [10, 11]. One exception is a preliminary report from our group, which found that a prominently high proportion of the evacuees experienced psychological distress [12]. Thus, in the present study, we performed a large-scale survey using the Fukushima Health Management Survey of 2011 (actually it was conducted in 2012) to clarify the actual mental health status of residents in the evacuation zone.

## Methods

### Study design

In 2012 within a year of the disaster, we analyzed data from the Mental Health and Lifestyle Survey as part of the Fukushima Health Management Survey, of which the primary purposes are to monitor the long-term health and daily lives of residents of Fukushima and to provide them appropriate care (S1 Appendix). The protocol of this survey is published in detail elsewhere[13].

### Participants

**Fig 1:**
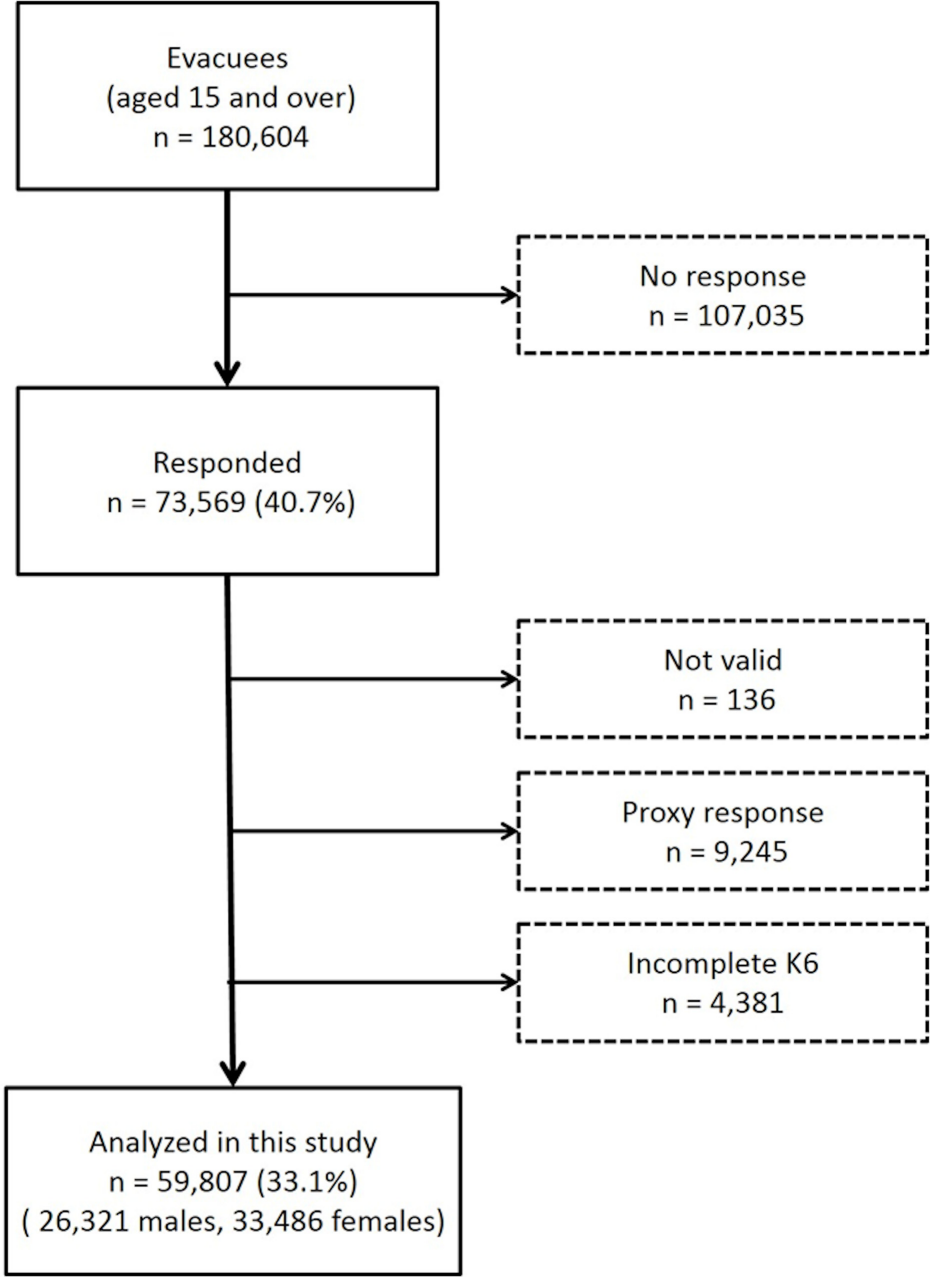
Flow of participant selection.

The target population for analysis was people 15 years and older as of March 11, 2011 who were all residents registered within the government-designated evacuation zone, which included the following municipalities: Hirono Town, Naraha Town,Tomioka Town, Kawauchi Village, Okuma Town, Futaba Town, Namie Town, Katsurao Village, Iitate Village, Minamisoma City, Tamura City, Kawamata Town and part of Date City in Fukushima Prefecture (n = 180,604, which was approximately 9% of all residents in Fukushima Prefecture in March 2011). Starting on January 18, 2012, we mailed self-administered questionnaires and asked the intended recipients or a proxy to mail back completed questionnaires (by October 31, 2012). Among them, 73,569 responses were returned, for a response rate of 40.7%. Out of the responses, 13,762 were excluded from this analysis; 136 failed to complete the questionnaires, 9,245 responded by proxy, and 4,381 had more than one missing value for the Kessler 6-item psychological distress scale (K6). In total, data from 59,807 participants were analyzed, for a valid response rate of 33.1% (Fig 1). Tables 1 and 2 show the baseline characteristics of participants which include those living in their own house who left the evacuation zone and returned. The analyses in this study were conducted anonymously. Written consent was obtained from respondents and guardians on behalf of the underage respondents upon enrollment in this study. This survey was approved by the ethics review committee of Fukushima Medical University (No. 1316).

**Table 1:** Participants’ demographic information and association with psychological distress (Kessler 6-item psychological distress scale, K6)^1^.

	Total, n	K6≥13, n (%)	
Total	59,897	8,717 (14.6)	
Gender			†
Male	26,321	3,133 (11.9)	
Female	33,486	5,584 (16.7)	
Age (years)			†
15–49	22,379	3,124 (14.0)	
50–64	19,315	2,874 (14.9)	
≥65	18,113	2,719 (15.0)	
Educational attainment			†
Elementary or Junior high school	12,415	1,966 (15.8)	
High school	29,755	4,385 (14.7)	
Vocational or Junior college	10,465	1,481 (14.2)	
University or Graduate school	5,243	573 (10.9)	
History of mental illness			†
Yes	2,865	1,228 (45.0)	
No	54,994	6,883 (12.5)	
Registered address on March 11, 2011			†
Hirono Town	1,394	202 (14.5)	
Naraha Town	2,285	380 (16.6)	
Tomioka Town	4,999	880 (17.6)	
Kawauchi Village	833	111 (13.3)	
Okuma Town	3,787	702 (18.5)	
Futaba Town	2,407	451 (18.7)	
Namie Town	7,674	1,444 (18.8)	
Katsurao Village	509	81 (15.9)	
Iitate Village	1,770	316 (17.9)	
Minamisoma City	19,884	2,883 (14.5)	
Tamura City	9,035	805 (8.9)	
Kawamata Town	4,365	374 (8.6)	
Part of Date City	865	88 (10.2)	

1:Chi-square tests were used.

†: *p*<0.01

**Table 2:** The association of different risk factors with psychological distress (Kessler 6-item psychological distress scale, K6)^1^.

Risk factors	Total, n	K6 ≥13, n (%)	
**Socioeconomic**			
Type of work			†
Full time	15,934	1,747(11.0)	
Part time	3,771	538(14.3)	
Unemployed^2^	26,787	4,291(16.0)	
Changed work situation^3^			†
Yes	31,461	5,676(18.0)	
No	25,327	2,466 (9.7)	
Started a new job			
Yes	1,070	183 (17.1)	
No	30,391	5493 (18.1)	
Became unemployed			†
Yes	12,722	2,581 (20.3)	
No	18,739	3,095 (16.5)	
Changed jobs			†
Yes	2,523	370 (14.7)	
No	28,938	5,306 (18.3)	
Income has increased			†
Yes	752	103 (13.7)	
No	30,709	5,573 (18.1)	
Income has decreased			†
Yes	11,366	1,966 (17.3)	
No	20,095	3,710 (18.5)	
**Disaster-related**			
Living place			†
In Fukushima prefecture	48,110	6,637 (13.8)	
Out of Fukushima prefecture	11,697	2,080 (17.8)	
Living arrangement			†
Evacuation shelter	525	100 (19.0)	
Temporary housing	5,438	937(17.2)	
Renting house, apartment	19,736	3,210 (16.3)	
Relative's house	2,199	330 (15.0)	
Own house	17,999	1,700 (9.4)	
Other	2,032	313 (15.4)	
Experience of Tsunami			†
Yes	12,032	2,251 (18.7)	
No	47,775	6,466 (13.5)	
Experience of Nuclear Power Plant Accident ^4^			†
Yes	31,366	5,754 (18.3)	
No	28,441	2,963 (10.4)	
Loss of someone close in the disaster			†
Yes	11,575	2,501 (21.6)	
No	47,091	5,963 (12.7)	

1:Chi-square tests were used.

†: *p*<0.01

2: Includes students and homemakers

3: Only the subjects who changed work situations replied to following socioeconomic questions (the new job, unemployment, job change, and income).

4: Defined as witnessing or hearing any hydrogen explosion at the nuclear power plant.

Note: Nuclear meltdowns at Fukushima Nuclear Power Plant caused several hydrogen explosions which occurred, the first in Unit 1, on 12 March and the last in Unit 4, on 15 March.

### Measures

We measured psychological distress as a primary outcome using the K6 [14]. The K6 is useful for epidemiological surveys detecting mood and anxiety disorders, as defined in the Diagnostic and Statistical Manual of Mental Disorders, Fourth Edition (DSM-IV). The areas under receiver operating characteristic curves (AUCs) were sufficiently high, as much as 0.94 (95% confidence interval [CI]: 0.88–0.99) [15]. In the K6, participants were asked if during the past 30 days they had felt “nervous”, “hopeless”, “restless or fidgety”, “so depressed that nothing could cheer you up”, “that everything was an effort” or “worthless”. Each question was rated on a 5-point Likert scale from zero (none of the time) to four (all of the time), with higher scores signifying worse mental health status (range: 0–24). The Japanese version of the K6 has been validated as an effective method for identifying psychological distress [16, 17].

We analysed only the questionnaires answered directly by the target cohort (n = 59,807). We dichotomized responders using a 12/13 cutoff on the K6 as those having psychological distress (≥13) and others (≤12) [17], and determined psychological distress for each risk factor [demographic information: gender (Male/Female), age (15-49/ 50-64/ ≥65 years), educational attainment (Elementary or Junior high school/ High school/ Vocational or Junior college/ University or Graduate school), history of mental illness (Yes/No), and registered address on March 11, 2011 including Hirono Town, Naraha Town, Tomioka Town, Kawauchi Village, Okuma Town, Futaba Town, Namie Town, Katsurao Village, Iitate Village, Minamisoma City, Tamura City, Kawamata Town, and A part of Date City; socioeconomic status: the type of work (Full time/ Part time/ Unemployed), changed work situation (Yes/No), started a new job (Yes/No), became unemployed (Yes/No), changed jobs (Yes/No), Change in income (increase, no cahnge, decrease); disaster-related factors: living place (In Fukushima prefecture/ Out of Fukushima prefecture), living arrangement (Evacuation shelter/ Temporary housing/ Renting house, apartment/ Relative's house/ Own house/ Other), experience of the tsunami (Yes/No) and the nuclear power plant accident (which was defined as witnessing or hearing any hydrogen explosion at the nuclear power plant; Yes/No), and loss of someone close in the disaster (Yes/No)]. In addition, we conducted bivariate analyses between mental health status and possible risk factors using the chi-square test. Further, to analyze the association between psychological distress and environmental radiation levels, we performed Spearman’s rank correlation between the proportion of those in the evacuation zone who scored ≥13 and the environmental radiation levels [In 2007, International Commission on Radiological Protection (ICRP) recommended a radiation dose limit for a normal period of 1 mSv/year (0.23 μSv/h) and of 20 mSv/year (4.6 μSv/h) for a restoration period] on a prefectural map of Fukushima based on the levels reported in a local newspaper Fukushima Minpo dated January 20, 2012 (in the beginning of this disaster, there had been no official data systematically obtained from both of Fukushima prefecture and the national government. At that time residents in Fukushima prefecture had no choice to read the data reported in a local newspaper in order to know their environmental radiation levels. This data used in our paper was measured by Fukushima prefecture or the national government and uploaded in their web pages. We decided to adopt this data because we thought that it effected on real-time psychological situation of residents at that time). Prevalence ratios (PRs) and 95% confidence CIs were estimated using modified Poisson regression models. We used modified Poisson instead of logistic regression because Poisson regression with robust variance can provide correct estimates and is a better alternative for the analysis of cross-sectional studies with binary outcomes than logistic regression, since the PR is more interpretable and easier to communicate to non-specialists than the odds ratio [18]. Adjustment variables consisted of age (≤49 years [reference], 50–64 years, ≥65 years; we divided age into 15–49 years, 50–64 years and ≥65 years the same as previous our study[19] because WHO defined ≧65 years as older or elderly person [(http://www.who.int/healthinfo/survey/ageingdefnolder/en/), and 15–49 years as reproductive age (http://www.who.int/reproductivehealth/topics/infertility/definitions/en/)], gender, living arrangement, experience related to tsunami and nuclear power plant accident, loss of someone close in the disaster, becoming unemployed (yes or no), decreased income (yes or no), history of mental illness (yes or no), and area [We grouped the eight areas (Hirono, Naraha, Tomioka, Okuma, Futaba, Namie, Katsurao, Iitate) together. These municipalities include those categorized as evacuation zones for all residents due to high dose radiation or those categorized as partial evacuation zones. The other five areas (Kawauchi, Minamisoma, Tamura, Kawamata and A part of Date) were excluded from the government-designated evacuation zones or partial evacuation zones]. We hypothesized that psychological distress would be influenced by above variables. Duplicate variables, such as increased income, change jobs in Table 2 may lead to over-adjusted so that we moved them from the model.

## Results

### The K6 scores of all subjects

The mean score for the K6 was 6.26 ± 5.75, and the median score, maximum score, minimum score, 25th percentile, and 75th percentile for the K6 were 5, 24, 0, 1, and 10, respectively. The number of those who scored ≥13 on the K6, meaning those with psychological distress, was 8,717 (14.6%) (Table 1).

### The association between the demographic, socioeconomic and disaster-related factors and psychological distress

**Fig 2:**
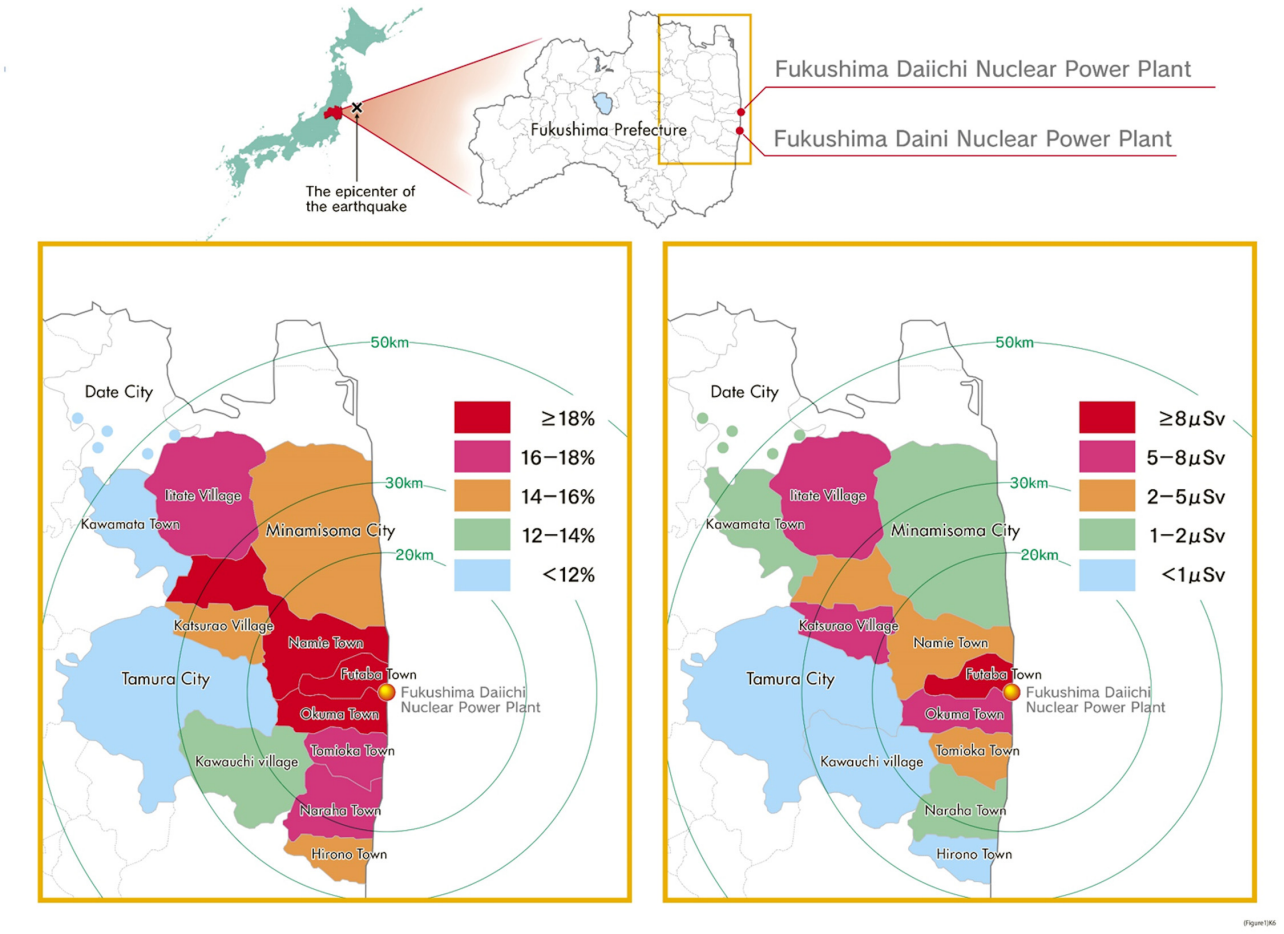
**The distribution of psychological distress in evacuation zone (A) and environmental radiation levels (μsV/h) in evacuation zone (B).** The distribution of psychological distress showed pattern similar to the environmental radiation levels on a prefectural map of Fukushima (based on the levels reported in a local newspaper Fukushima Minpo dated January 20, 2012). Spearman’s rank correlation showed that the proportion of those in the evacuation zone who scored ≥13 on the K6 was significantly highly correlated with the environmental radiation levels (*r* = 0.768, *p* = 0.002). The 18% in (A) means the area where more than 18% of the participants scored >13 on the K6, and >8 μSv in (B) means the area where >8 μSv/h was recorded. Original maps were created by tracing copyright-free materials (http://kage-design.com/wp/?p=1061 and http://www.civilcom.co.jp/library/WhiteMapJapan/#07) and then drawing the content using Adobe Illustrator CS6 (Adobe Systems Inc., San Jose, CA).

Table 2 demonstrates the demographic information, socioeconomic status, disaster-related factors of the participants, and the results of chi-square tests examining the associations between psychological distress and each factor in Tables 1 and 2. For demographic factors or socioeconomic and disaster-related factors, we found significant differences in the prevalence of psychological distress by almost all survey items. we found significant differences in the prevalence of psychological distress by demographic information: gender (males, 11.9%; females, 16.7%, *p*<0.01), age (15–49, 14.0%; 50–64, 14.9%; ≥65, 15.0%, *p*<0.01), educational attainment (elementary or junior high school, 15.8%; high school, 14.7%; vocational or junior college, 14.2%; university of graduate school, 10.9%, *p*<0.01), history of mental illness (yes, 45.0%; no, 12.5%, *p*<0.01), and registered address on March 11, 2011 (Hirono Town, 14.5%; Naraha Town, 16.6%; Tomioka Town, 17.6%; Kawauchi Village, 13.3%; Okuma Town, 18.5%; Futaba Town, 18.7%; Namie Town, 18.8%; Katsurao Village, 15.9%; Iitate Village, 17.9%; Minamisoma City, 14.5%; Tamura City, 8.9%; Kawamata Town, 8.6%; Part of Date City, 10.2%, *p*<0.01) in which Namie, Futaba and Okuma Towns are higher and Kawamata Town is the lowest (Fig 2). For socioeconomic status, we found significant differences in the prevalence of psychological distress by the type of work (full time, 11.0%; part time, 14.3%; unemployed, 16.0%, *p*<0.01), changed work situation (yes, 18.0%; no, 9.7%, *p*<0.01), became unemployed (yes, 20.3%; no, 16.5%, *p*<0.01), changed jobs (yes, 14.7%; no, 18.3%, *p*<0.01), income has increased (yes, 13.7%; no, 18.1%, *p*<0.01), and income has decreased (yes, 17.3%; no, 18.5%, *p*<0.01). In disaster-related factors, we found significant differences in the prevalence of psychological distress by living place (in Fukushima Prefecture, 13.8%; out of Fukushima Prefecture; 17.8%, *p*<0.01), living arrangement (evacuation shelter, 19.0%; temporary housing, 17.2%; renting house/apartment, 16.3%; relative’s house, 15.0%; own house, 9.4%; other, 15.4%, *p*<0.01), experience of tsunami (yes, 18.7%; no, 13.5%, *p*<0.01) and the nuclear power plant accident (yes, 18.3%; no, 10.4%, *p*<0.01), and loss of someone close in the disaster (yes, 21.6%; no, 12.7%, *p*<0.01).

Furthermore, after adjustment for confounding variables, female gender, older age, experience of the nuclear power plant accident, loss of someone close in the disaster, unemployment, history of mental illness, and area were associated with increased PRs (Table 3). Compared with those living in a relatives' home or their own home, the participants living in a rental house or apartment and evacuation shelter or temporary housing were associated with increased PRs, while the PR was higher for those living in a rental house or apartment than those living in an evacuation shelter or temporary housing.

**Table 3:** Multivariable-adjusted prevalence ratios (PRs) and 95% confidence intervals (Cis) for psychological distress on modified Poisson regression analyses.

		Total
Variables		PR^1^ (95%CI)
Gender	Male (Reference)	1.00 (reference)
	Female	1.88 (1.72–2.06)
Age	15–49 (Reference)	1.00 (reference)
	50–64	1.12 (1.01–1.24)
	> = 65	1.18 (1.01–1.38)
living arrangement	Relatives' home or own home (Reference)	1.00 (reference)
	Rental house, apartment	1.47 (1.25–1.73)
	Evacuation Shelter or Temporary housing	1.21 (1.12–1.32)
Experience of Tsunami	No (reference)	1.00 (reference)
	Yes	1.13 (1.07–1.19)
Experience of the nuclear power plant accident	No (reference)	1.00 (reference)
	Yes	1.48 (1.41–1.55)
Loss of someone close in the disaster	No (reference)	1.00 (reference)
	Yes	1.41 (1.34–1.48)
Unemployment	No (reference)	1.00 (reference)
	Yes	1.25 (1.19–1.32)
Decreased income	No (reference)	1.00 (reference)
	Yes	1.20 (1.14–1.26)
Mental disorder history	No (reference)	1.00 (reference)
	Yes	10.18 (9.01–11.51)
Area	Others (reference)	1.00 (reference)
	Hirono, Naraha, Tomioka, Okuma, Futaba, Namie, Katsurao, Iitate	1.21 (1.15–1.27)

^1^Adjustment for age, gender, living arrangement, experience of the nuclear power plant accident, loss of family member, unemployment, history of mental disorder, and area.

### The distribution of psychological distress according to evacuation zone and environmental radiation levels

We presented the distribution of psychological distress according to environmental radiation levels on a prefectural map of Fukushima (Fig 2). The proportions of those in the evacuation zone who scored ≥13 on the K6 were significantly positively correlated with the above-mentioned environmental radiation levels (*r* = 0.768, *p* = 0.002).

## Discussion

We described in detail the mental health status of residents in the evacuation zone around the Fukushima Daiichi Nuclear Power Plant. To the best of our knowledge, this article presents the first large-scale and systematic study to elucidate the characteristics of the mental health status of these residents. There have been no reports on individual risk factors for psychological distress, including demographic information, socioeconomic variables, and disaster-related variables in the early stage after the nuclear disaster. Although there have been studies on the Three Mile Island (TMI) accident and the Chernobyl nuclear power plant disaster, the detailed analysis of the mental health of those living in Fukushima Prefecture is crucial because both the TMI accident and Chernobyl disaster arose singly without the combination of other natural disasters, and in the near future similar complex disasters involving a nuclear accident or nuclear terrorism might occur somewhere in the world. Therefore, we believe that our findings may benefit mental health professionals working with after nuclear disaster survivors.

In the present study, the proportion of psychological distress in the evacuation zone was markedly high, at 14.6%. Kawakami described in a Japanese report [20] that the proportion of residents who scored above the cutoff point of 13 on the K6 was 3.0% in non-disaster periods in Japan, suggesting the severity of the mental health status of residents in the evacuation zone in Fukushima although we should consider the potential differences in the baseline characteristics between the two study populations and have to interpret this comparison carefully. We found that basic demographic factors including age and sex were significantly associated with mental health state; that is, psychological distress increased with age, and was higher in females than males. These age and gender differences in the psychological burden of the disaster are consistent with previous studies that analyzed psychological burden after an earthquake, hurricane, or tsunami [21, 22]. On the other hand, in the other regions affected by the Great East Japan Earthquake and subsequent tsunami but not the nuclear power plant accident, such as Iwate Prefecture, various variables, for instance, female gender, younger male, health complaints, severe economic status, relocations, and lack of a social network, were associated with poor mental health [23]. However, the proportion of psychological distress in this population was 6.2%, which was significantly lower than the one in our cohort (14.6%), based on the chi-square test (*p*<0.001). Since we have just compared the proportion of mental distress in both residents and did not compare the details, we should take this remarkable gap cautiously considering the potential differences in the baseline characteristics between the two study populations.

For other demographic factors, Educational attainment, History of mental illness and area (Registered address on March 11, 2011) were significantly associated with psychological distress in our results. Higher education levels decreased the risk of psychological distress, which is consistent with previous studies on disasters [24] [25]. In accordance with several studies on changes in mental status among psychiatric patients following the nuclear accident [10, 11] [26], having mental illness was highest risks in our all survey items. Living area will be discussed below. For socioeconomic factors, almost all items were significantly associated with psychological distress in our population. It was also supported by previous study [23] which suggested that severe economic status may be important risk factors of poor mental health and emphasized the importance of economic support and employment, particularly for men. However, the difference seen in “Income has decreased” was unexpected, as we thought that those who answered yes might be more psychological distressed than those who answered no. It is difficult to interpret this result. For disaster-related factors, Living place, Living arrangement, experience of tsunami or the nuclear power plant accident and loss of someone close in the disaster were significantly associated with psychological distress. After Hurricane Katrina, post-traumatic stress disorder (PTSD), psychological distress and suicide attempts increased with time, and psychological distress increased among respondents not living in the same town as before the hurricane, compared to those living in the same town[27]. These results are consistent with our results showing that psychological distress for those living outside Fukushima Prefecture after the disaster was significantly higher than those living in Fukushima Prefecture (17.8% and 13.8%, respectively). Also, several previous studies [28] [23] suggested that relocation after a disaster increased psychological distress, which was supported by our finding. Disaster-related experiences such as experience of tsunami or the nuclear power plant accident and loss of someone close in the disaster were also related to psychological distress in this population as already reported by other previous studies [29] [30]. Above all, the experience of the nuclear power plant accident was distinctive feature in this complex disaster.

To date, there have only been two nuclear power plant accidents: the TMI accident in the United States and the Chernobyl nuclear power plant disaster in the former Soviet Union. Several studies on mental health after the TMI and Chernobyl accidents have been reported [8, 31–33]. Many experts have recognized that the effect of the Chernobyl accident on mental health was one of the largest public health issues at the time. Stress-related symptoms including depression, anxiety, PTSD, and medically unexplained somatic symptoms were elevated in the cohort of the Chernobyl disaster compared to controls[8] [31]. These consequences are often related to fears about developing malignant neoplasm caused by exposure to excess radiation, and evidence from the Chernobyl or TMI accidents suggests that females with young children and cleanup workers at nuclear power plants have the highest risk of suffering from psychological distress, even if not directly exposed to radioactive contamination[32]. There is confirmed evidence that mental health problems were significantly elevated following the TMI and Chernobyl accidents. Meanwhile, the Fukushima disaster was a complex series of events that human beings have never before experienced—an earthquake, tsunami, and nuclear power plant accident. Therefore, given the unprecedented and devastating nature of this disaster, we would expect that psychological distress would be highly prevalent in the residents in the evacuation zone around the Fukushima Daiichi Nuclear Power Plan, as was found in studies of other nuclear disasters, such as TMI and Chernobyl accidents.

The results of the present study already show that the living area (Registered address on March 11, 2011) was significantly related to psychological distress. Moreover, we found that the distribution of psychological distress exhibited a pattern similar to that of the environmental radiation levels in Fukushima Prefecture. In fact, these two factors were significantly highly correlated. It can be interpreted that the mental health of the residents was associated with the radiation levels in the government-designated evacuation zones, and the mental health of the residents might also be modulated by various socioeconomic and disaster-related factors (female gender, older age, living arrangement, experience of the nuclear power plant accident, loss of someone close in the disaster, unemployment, history of mental illness, and area) on the basis of multivariate analyses in this study. Moreover, the mental health of evacuees might be further modulated by their exacerbated risk perception of radiation health effects. We have reported the relationships between psychological distress and its associated factors in relation to risk perception of radiation health effects among the evacuees of Fukushima Prefecture in another paper [19]. This correlation analysis is preliminary, however, and more detailed analyses of the association between psychological distress and reported environmental radiation levels are needed.

Incidentally, another study found that the proportion of psychological distress in workers at the Fukushima Nuclear Power Plants was exceedingly high (Daiichi,47%; Daini, 37%) [29], which also indicates the serious impact of a nuclear accident on mental health. In Fukushima, looking over the entire course of the disaster, the emergency attempts to reduce the environmental radiation levels, emergency medical care, and general medical services were adequate, despite the severity of this unprecedented disaster. Nonetheless, there is strong concern that residents in Fukushima Prefecture are at high risk for psychological distress, much like residents near the Chernobyl accident. Therefore, understanding not only mental health accompanied with their risk perceptions, as reported in the TMI and Chernobyl accidents, but also mental health from the standpoint of the social impact is critical.

There are several strengths and limitations in this study. Firstly, this study was a large-scale and systematic survey among evacuees who experienced complex events of an earthquake, tsunami, and nuclear accident. Although this study has such a strength, we could not provide enough useful data to delineate the differential impacts of natural disaster and nuclear power plant disaster. Secondly, this study collected data within one year after the disaster occurred. However, this study was conducted in a restricted region in Japan, so the external validity of these findings is limited. In addition, we should consider common method bias because this would inflate the associations of a high K6 score with the demographic, socioeconomic, and disaster-related factors. If data on dependent and independent variables were collected in the same or similar method, their associations can be inflated and this increases the risk of the type I error [34]. We also have to consider selection bias since the representativeness of these results is limited by the valid response rate of only 33.1%. Thus, there are possible differences between those who did and did not respond to the questionnaire and their effects on the results. One possible difference would be that mentally distressed evacuees more often responded for appeal of and help for their psychological distress than mentally healthy evacuees. Furthermore, since this study was a cross-sectional study, causal relationships between psychological distress and socioeconomic factors were not clear. However, the psychological burden of the disaster-related factors, such as living arrangement and experience related to the nuclear accident, might be inferable. We need to conduct further analyses, for example, combining the two variables “experience of tsunami” and “experience of nuclear power plant” into one variable and then doing stratified analyses. Lastly, although the K6 scale is useful in epidemiological surveys to screen those with mood and anxiety disorders, psychological distress of the evacuees cannot be fully assessed by a single use of the K6 scale.

## Conclusions

The large-scale earthquake and tsunami followed by the accident at the Fukushima Daiichi Nuclear Power Plant likely caused severe psychological distress among residents in the evacuation zone in Fukushima Prefecture. Considering the degree and continuity of the negative influences on the mental health of the general population, long-term surveys, close observations should be continued. Moreover on the basis of the results in this study psychosocial interventions for evacuees, especially for female gender, older age, living in evacuation shelter, living outside Fukushima Prefecture after the disaster, experience of the nuclear power plant accident, loss of someone close in the disaster, history of mental illness, and Registered address on March 11, 2011 where the environmental radiation level was higher, are strongly recommended.

## Supporting Information

S1 Appendix The data we analyzed in this study from the Mental Health and Lifestyle Survey as part of the Fukushima Health Management Survey. (XLSX)
